# Improving results in rat fracture models: enhancing the efficacy of biomechanical testing by a modification of the experimental setup

**DOI:** 10.1186/s12891-018-2155-y

**Published:** 2018-07-19

**Authors:** Peter Michael Prodinger, Dominik Bürklein, Peter Foehr, Kilian Kreutzer, Hakan Pilge, Andreas Schmitt, Rüdiger v. Eisenhart-Rothe, Rainer Burgkart, Oliver Bissinger, Thomas Tischer

**Affiliations:** 1 Klinik für Orthopädie und Sportorthopädie, Klinikum rechts der Isar der Technischen Universität München, Ismaninger Straße 22, 81675 Munich, Germany; 2 Abteilung für Fuß- und Sprunggelenkchirurgie, Klinik Volkach, Volkach, Germany; 3 Abteilung für Biomechanik, Klinik für Orthopädie und Sportorthopädie, Klinikum rechts der Isar der Technischen Universität München, Munich, Germany; 4 Klinik für Mund-, Kiefer- und Gesichtschirurgie, Universitätsklinikum Hamburg Eppendorf, Hamburg, Germany; 5 Orthopädische Klinik, Universitätsklinikum Düsseldorf, Heinrich-Heine-Universität, Düsseldorf, Germany; 6 Abteilung für Sportorthopädie, Klinik für Orthopädie und Sportorthopädie, Klinikum rechts der Isar der Technischen Universität München, Munich, Germany; 7 Klinik für Mund-, Kiefer- und Gesichtschirurgie, Klinikum rechts der Isar der Technischen Universität München, Munich, Germany; 8 Orthopädische Klinik und Poliklinik der Universität Rostock, Rostock, Germany

**Keywords:** Rat fracture studies, Three-point bending, Biomechanical testing, Animal model, pQCT

## Abstract

**Background:**

Animal fracture models, primarily performed in rats, are crucial to investigate normal and pathological bone healing. However, results of biomechanical testing representing a major outcome measure show high standard deviations often precluding statistical significance. Therefore, the aim of our study was a systematical examination of biomechanical characteristics of rat femurs during three-point bending. Furthermore, we tried to reduce variation of results by individually adapting the span of bearing and loading areas to the bone’s length.

**Methods:**

We examined 40 paired femurs of male Wistar-rats by DXA (BMD and BMC of the whole femur) and pQCT-scans at the levels of bearing and loading areas of the subsequent biomechanical three-point bending test. Individual adjustment of bearing and loading bars was done respecting the length of each specimen. Subgroups of light (< 400 g, *n* = 22) and heavy (> 400 g, *n* = 18) animals were formed and analysed separately. We furthermore compared the results of the individualised bending-setting to 20 femurs tested with a fix span of 15 mm.

**Results:**

Femurs showed a length range of 34 to 46 mm. The failure loads ranged from 116 to 251 N (mean 175.4 ± 45.2 N; heavy animals mean 221 ± 18.9 N; light animals mean 138.1 ± 16.4 N) and stiffness ranged from 185 N/mm to 426 N/mm (mean 315.6 ± 63 N/mm; heavy animals mean 358.1 ± 34.64 N/mm; light animals mean 280.8 ± 59.85 N/mm). The correlation of densitometric techniques and failure loads was high (DXA R^2^ = 0.89 and pQCT R^2^ = 0.88). In comparison to femurs tested with a fix span, individual adaptation of biomechanical testing homogenized our data significantly. Most notably, the standard deviation of failure loads (221 ± 18.95 N individualized setting vs. 205.5 ± 30.36 N fixed) and stiffness (358.1 ± 34.64 N/mm individualized setting vs. 498.5 ± 104.8 N/mm fixed) was reduced by at least one third**.**

**Conclusions:**

Total variation observed in any trait reflects biological and methodological variation. Precision of the method hence affects the statistical power of the study. By simply adapting the setting of the biomechanical testing, interindividual variation could be reduced, which improves the precision of the method significantly.

**Electronic supplementary material:**

The online version of this article (10.1186/s12891-018-2155-y) contains supplementary material, which is available to authorized users.

## Background

Preclinical research concerning normal and disturbed bone healing is usually done in small animal models [1–3]. Of those, rat fracture models are of paramount importance [4]. Still, a significant proportion (38%) of all published animal fracture studies used rats for their investigations [5]. Furthermore, they are useful to advance tissue-engineering methods and to characterize positive or negative effects of certain types of medication on bone healing that have been observed or suspected in the clinical use [6, 7].

Analysis of results is carried out by morphological and functional investigations. Regularly applied radiological procedures such as DXA, pQCT and Micro-CT provide detailed analyses of the bone microstructure [6, 8, 9]. In addition to bone density and mineral content, the raw data allows for a calculation and comparison of geometric parameters. Histological examinations enable cellular analyses and even dynamics in bone apposition can be visualized by fluorescence staining [10]. Of all the analytical methods mentioned – no matter how advanced and elaborated they might be - only functional trials using biomechanical testing provide answers to the most important question: the stability of bone [11].

Over the last few years, several experimental studies have been carried out on bone healing in animals under pharmacological influence [12–16]. While most of the studies showed significant differences in volume, formation and mineralization of the callus, these findings could often not be confirmed biomechanically. This led us to the question whether the apparent morphological variability is, in fact, irrelevant - or if the applied testing protocols are simply not good enough to measure small effects. In monitoring imaging technique developments over the past decades, the array of possibilities and their accuracy have expanded considerably [7, 17]. As a result, smaller effects could undoubtedly be verified through imaging. By contrast, biomechanical testing has not shown comparable progress.

Animal models often show a wide range of standard deviations in biomechanical testing protocols, leading to non-significant outcomes. Therefore, the aim of our study was to reduce variability and to obtain statistically sound results by doing a systematic examination of biomechanical characteristics of rat femora tested in an individualized setting. Furthermore, we think that there is a need for standardization of biomechanical testing procedures in small animal studies [18].

## Methods

### Animals

The study was performed in the animal facility of the Klinikum rechts der Isar, Technische Universität München and was evaluated and approved by the local authority (Regierung von Oberbayern, approval no. 55.2–1–54-2531-17-10) as required by German law.

As described previously [19], 20 male Wistar rats (individual testing setup) with a mean body weight of 476 ± 160 g (range between 240 and 720 g) and another 10 male Wistar rats (fixed testing setup) with a mean body weight of 634.5 ± 26.9 g (range between 585 and 679 g) were euthanised according to the recommendation of the European Union using a threefold overdose of Phenobarbital (90 mg/kg) i.v. Subsequently the extremities were dissected and both femurs (*n* = 60) harvested. Surrounding skin, muscle, and other soft tissues were removed. The bones were fixed and stored in 70% Methanol at 4 °C.

For further analysis, the individual testing setup group was divided into subgroups of animals below and above 400 g. Specimens below 400 g corresponded to young animals under 16 weeks, above 400 g to adults above 16 weeks of age.

### High resolution dual energy X-ray absorptiometry (DXA)

The methodology of the radiological biomechanical analyses has been described before [19].

Bone mineral content (BMC) and lean mass was measured in all femurs of the individual testing group (*n* = 40) using a high resolution DXA scanner (pDEXA Sabre, Norland/Stratec, Stratec Medizintechnik, Pforzheim) [20]. The resolution was 100 μm × 100 μm (acquisition time: 5 mm/s, total time 40 min). By DXA the bone mass can be normalized to the surface area of the bones exposed to radiation (bone mineral density = BMD in in mg/cm^2^), whereas analysis of the volumetric bone density (mg/cm^3^) is not possible. This results in a higher BMD for bigger bones but not necessarily in discrepancies in volumetric density [21].

Immediately after DXA scanning, the length of the bones, as well as the anterior-posterior diameter at the middle of the diaphysis were assessed with a calliper.

### Peripheral quantitative computed tomography (pQCT)

All femurs of the individualized bending setup (*n* = 40) were examined using a pQCT scanner (XCT Research SA+, Stratec Medizintechnik, Pforzheim). Before the measurement, excessive air was removed in a vacuum chamber (20 min). After a quick-view (Scout-View), transverse section images with a thickness of 500 μm and a spatial resolution within the image plane of 100 μm at the diaphysis and the distal and proximal metaphysis were acquired. Based on the scout-view, each bone’s individual length was designated 100%. Measurement positions were defined at the distal femur metaphysis at 15, 17.5 and 20%, at the middle of the femur diaphysis at 40, 42.5 and 45% and proximal at 64, 66.5 and 69% of the femoral bone length [19]. The average value was calculated from the three cross section images at all sites. Positioning of bearing- and loading-bars during the subsequent three-point bending test corresponded to the pQCT transection positions.

Further analysis of the qualitative variables and evaluation of the cross-sections was done with the software provided by the manufacturer. We used peelmode 1 with a threshold of 600 mg/mm and peelmode 2 (40% surface with lowest density = trabecular compartment) to enable separate calculations of trabecular, cortical and subcortical bone properties [22].

The cortical mineral content, the cortical density and thickness of the cortical bone, the cortical bone cross section surface and its percentage of the whole cross section surface were assessed at the middle of the diaphysis. All parameters are supposed to indicate mechanical strength of the bone during bending. Furthermore, the area surface moment of inertia (IP), the moment of resistance, as well as minimal and maximum moment of inertia were determined.

### Mechanical testing (Fig. 1)

**Fig. 1:**
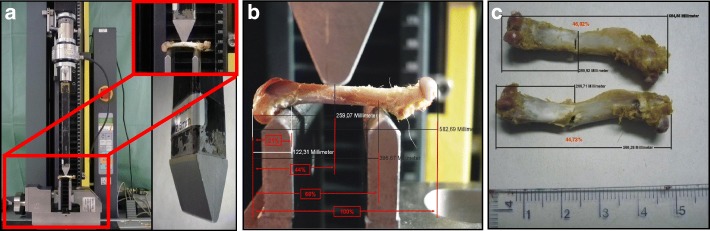
Biomechanical Testing. **a** Setup of three-point bending with individual adjustment of breaking and loading bars. Individual adaption of the loading bars is facilitated by a helical wheel (left lower corner). The apparatus is relocatable to keep the breaking bar centred. **b** Individual adjustment of breaking and loading bars are identical to pQCT measurements. Every specimen was placed onto the flat area of the poplitheal plane (distal supporting bar), which is oriented in the frontal plane anatomically. Contact areas on the supporting bars were point-shaped proximally (just below the lesser trochanter) and either linear-shaped or two point-shaped (in case of prominent tails of the linea aspera) distally. This achieved a stable triangular-shaped bearing under loading, preventing rotational movements during the three-point bending test and standardizing the ap-orientation of the bone. **c** Rat femurs after mechanical testing, fracture occurred accurately at the predicted percentage

After rehydration in saline solution, the bones were loaded until failure with a persistent test velocity of 5 mm/min (three-point setup, AP direction) using a Zwick material testing machine (Zwicki Line Z2.5, Zwick GmbH & Co, Ulm, Germany). Concerning the individualized setup group, the position of the supporting- and loading-bars corresponded to the pQCT assessment areas, with the force transmission at the evaluated middle position between the distal and proximal site. The distance between the bars was adapted relative for each bone, the bearing bars were adjusted between 15 and 20% distal and 64 to 69% of each femurs length proximal, the loading bar in the middle (40–45%, see Fig. 1). The tips of the bars were rounded with a diameter of 2.5 mm. A load-displacement diagram was recorded every 0.1 s during bending; thereby the failure load could be determined. We measured the reaction forces with a load cell for up to ±2.5 kN (Klasse 0.05, A.S.T. GmbH, Dresden, Germany). Stiffness was defined as linear regression of the force and displacement graph (testXpert V12, Zwick/Roell, Ulm, Germany).

The 20 femurs of the fixed setup group were tested biomechanically with a fix span of 15 mm between the supporting bars.

### Statistical analysis

Acquired data was analysed using GraphPad Prism 5 (GraphPad Software, La Jolla, CA, USA). Descriptive statistics are given by mean ± standard deviation, as normal distribution could be assumed by the distribution of the single values. Percentage variation (CV%) was calculated as CV% = SD/Mean * 100. Group comparisons were performed by Student’s t-test. *P*-values < 0.05 were declared as statistically significant different. The correlations were determined by the slope of linear regression graph (including the coefficient of determination R^2^ and the 95% confidence intervals).

## Results

### Individualized bending setup

Femurs measured provided a length between 34 and 46 mm. Both femurs of one individual were very constant; especially in light animals, barely no side differences were evident. Subgroup analysis showed that the femoral length of light rats (< 400 g) was 35.5 ± 1.3 mm, heavy rat’s (> 400 g) mean was 43.1 ± 1.6 mm.

The failure loads of all specimens ranged from 116 to 251 N (mean 175.4 ± 45.2 N), heavy animals had a mean of 221 ± 18.95 N; light animals showed a mean of 138.1 ± 16.38 N. The stiffness of all specimens showed a larger range of results than the failure loads (Min 185 N/mm, Max 426 N/mm; Mean 315.6 ± 62.6 N/mm) (see Table 1).

**Table 1:** Summary, biomechanical data, individualized-setting group

	Total	Light (< 400 g)	Heavy (> 400 g)	Light vs. Heavy
	*N* = 40	*N* = 22	*N* = 18	
Lenght (mm)	38.9 (±4.1)	35.5 (±1.3)	43.1 (±1.6)	*p* < 0.001
Failure load (N)	175.4 (±45.2)	138.1 (±16.38)	221.0 (±18.95)	p < 0.001
Stiffness (N/mm)	315.6 (±63.0)	280.8 (±59.85)	358.1 (±34.64)	*p* = 0.0003

Means and standard deviations of length, failure load and stiffness. Total and subgroups of light and heavy animals separately

Pairing of specimens (left vs. right) did not result in statistically significant differences altogether. However, we observed length differences of more than 1 mm in six of nine heavy animals (whereas in small animals both bones were almost equal). Furthermore, six of nine heavy rats showed a stronger right femur concerning the failure loads (which was not necessarily the longer bone, difference between the two groups still not statistically significant: *p* = 0.072), the left/right ratio in failure loads of small animals was almost balanced (5/6 in favor of right bones).

**Fig. 2:**
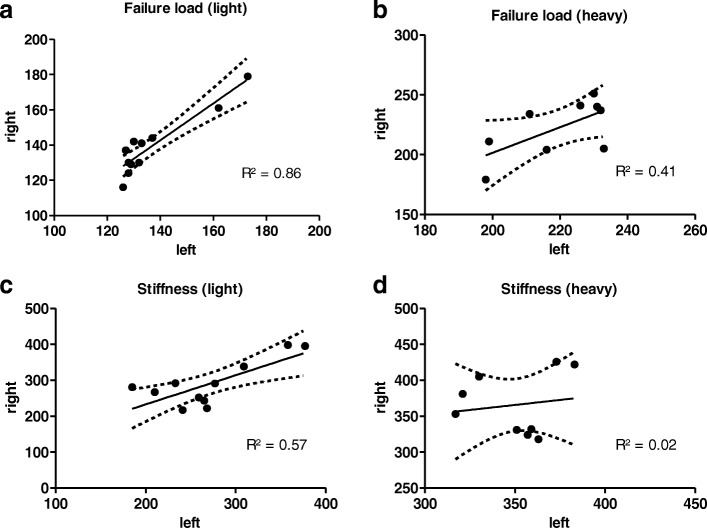
Correlation graphs of failure loads and stiffness values, right versus left femur of each specimen (Bivariate Scattergrams with regression lines and 95% confidence bands). **a**, **b** Failure loads of the right versus the left femur. Light rats (> 400 g, **a**) show a strong correlation. In heavy animals (> 400 g, **b**) a wide range between both sides was evident. **c**, **d** Stiffness values of the right versus the left sides. In light and heavy animals, no correlations between both sides could be seen

Interindividual variance (CV%) in failure load was 25.8% in all specimens and could be significantly reduced to under 9% if light animals were excluded, or 11.9% if heavy animals were excluded. The correlation of failure loads between the left and right side was higher in light animals (R^2^ = 0.86), in heavy animals the coefficient of determination between the left and right side was only 0.4. Concerning the stiffness, a relatively weak correlation between both sides was observed either in light as in heavy specimens (see Fig. 2).

**Fig. 3:**
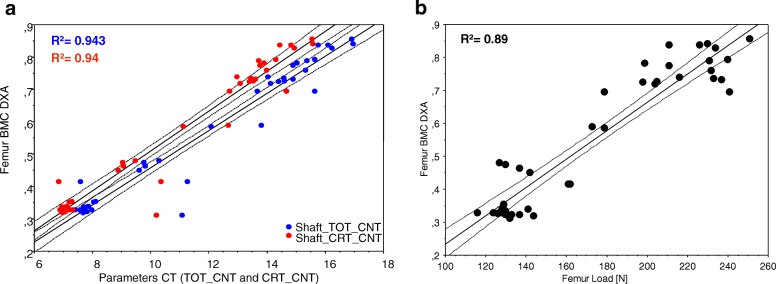
Correlation graphs (Bivariate Scattergrams with regression lines and 95% confidence bands). **a** Femoral DXA BMC (bone mineral content in g) versus pQCT total content (TOT_CNT, blue dots) and cortical content (CRT_CNT, red dots) show a strong correlation of both techniques. **b** Femoral DXA BMC (bone minerall content in g) versus failure load R^2^ = 0.89. The correlation between pQCT – values (TOT_CNT and CRT_CNT) is identical

Over all, both densitometric techniques (DXA, pQCT) showed a high correlation between each other (R^2^ = 0.94) (see Fig. 3a). The correlation of the densitometric techniques and the failure loads reached consistent and high values (DXA R^2^ = 0.89 and pQCT R^2^ = 0.88) (see Fig. 3b). The percentual deviation by the DXA for the BMD (left vs. right) was 0.6 ± 2,3% (BMC 1.5 ± 4.5%), by CT 2.5 ± 5.3% (n.s.).

### Comparison individualized bending setup vs. fix span

**Fig. 4:**
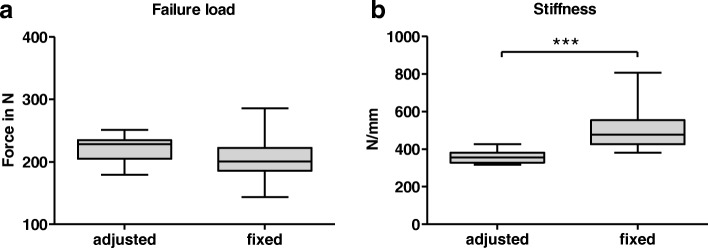
Box Plots, Failure loads and Stiffness, adjusted (inidvidualized) setting vs. fixed span during three-point bending. **a** Failure loads of individual (adjusted) vs. fixed (15 mm) setting during three-point bending. A strong tendency (*p* = 0.07) to obtain different results by altering the setup was evident. Furthermore, the standard deviation within the sample was lowered by individual adjusted loading bars (18.95 N vs. 30.4 N, see Table 2). **b** Stiffness values of individual (adjusted) vs. fixed (15 mm) setting during three-point bending. Highly significant difference (*p* < 0.0001), the standard deviation of the adjusted sample was only about one third compared to fixed bars (34.6 N/mm vs. 104.8 N/mm)

In comparison to femurs tested with a fix span, individual adaptation of biomechanical testing improved the results significantly (see Table 2). Failure loads were higher using the individualized approach (mean 221 N adjusted setup vs. 205.5 N fixed span) and the standard deviation could be reduced by one-third (18.95 N adjusted setup vs. 30.36 N fixed). Concerning stiffness measurements, the changes caused by the treatment protocol appeared even bigger. By adapting the bars to the individual bones length, stiffness was reduced to a mean of 358.1 ± 34.6 N/mm, while femurs tested with a fix span showed a mean of 498.5 ± 104.8 N/mm. Besides reaching different absolute values, a diminution of the variation within the trait by individualized bending was feasible (see Fig. 4). Every single biomechanical and radiological measurement is depicted in the additional data sheet (Additional file 1).

**Table 2:** Summary, biomechanical data, comparison between fixed and individualized bending setup

	Fixed Span	Individualized	Fixed vs. Individualized
	*N* = 20	*N* = 18	
Weight (g)	634.5(±26.9)	641.9 (±55.5)	*p* = 0.59
Length (mm)	42.9(±1.1)	43.1(±1.6)	*p* = 0.63
Failure Load (N)	205.5 (±30.36)	221.0 (±18.95)	*p* = 0.07
Stiffness (N/mm)	498.5 (±104.8)	358.1 (±34.64)	*p* < 0.0001

Means and standard deviations of weight, length, failure load and stiffness. Subgroups of femurs tested with a fixed span and heavy animals tested by the individualized setting. Both groups are not statistically significant different concerning weight or length. Though the fixed-span subgroup seems more homogenous concerning weight and lengths of the femurs, failure loads (tendency) and stiffness values (stat. significant) are more homogenous in the individualized-setting group

## Discussion

Accurate comparisons of biomechanical studies concerning bone strength are often impaired by lacking standardisation of the biomechanical testing protocols. Published techniques have included tensile testing, compressive loading, torsional testing, four-point bending and three-point bending tests [4, 23]. Of these, three-point bending is a simple, reproducible test, making it the preferred method of mechanical testing in small animal fracture models [4, 8, 24]. Usually, the three-point bending setup implies bearing bars with a fixed distance of 14 to 18 mm and a loading intender centred in between (regardless of individual bone length); force is applied perpendicularly to the bone with a constant speed of 2 to 5 mm/min [13, 25]. So even a simple procedure like three-point bending has experienced multiple modifications precluding comparability and integrity of data.

Preclinical animal models using rats have a high prognostic value concerning the efficacy and safety of drugs in humans [3, 6, 26]. Because the cellular phenotype of osteoblasts and osteoclasts is comparable, the effect of medication on bone and fracture healing in rats allows deductions about the human situation, designating these models ideal in exploring unknown or adverse effects on bone. However, the absence of the Haversian system and the presence of permanently open growth plates in rodents indicate fundamental discrepancies and prevents direct extrapolation of the results to humans [26]. Both, tibiae and femurs of rats are suitable for experimental studies. The tibia is surrounded by a thin soft tissue mantle allowing easy access to the bone and favouring this bone for closed fracture models without surgery [27]. Thus, the risk of generating concomitant fibular fractures is high and changes a stable into an unstable model, potentially implicating the need for secondary surgical stabilisation with a significant amount of complications [28]. The femur on the other hand is surrounded by a thick muscle layer impeding the generation of standardized fractures, but allowing the use of proper implants with acceptable complication rates. Furthermore, most, widely used fracture models are established regarding the femur offering the possibility for comparisons between different studies. To summarize, most authors would prefer the femur when fracture healing is the aim of the study [29].

Reviewing the recent literature, we surprisingly found that systematic tests/research on inter- and intra-individual differences of rat femurs are, for the most part, not available. Bones come in different shapes and sizes, and none have the geometry and gross morphology of an ideal mechanical test specimen. Hence, adapting the biomechanical testing setup to each bone individually seems a logical step to generate results that are more reliable. First and foremost the length of tested bones shows great impact on failure loads. As approximations of the failure/braking load are displayed by F = 48*E*I*d/L^3^ (F = breaking load, E = Young’s modulus, I = second moment of area, L = length between the supporting bars, d = deformation), naturally, modifications of the distance between the supporting bars provides major impact on the actual failure load [9]. By comparing failure load and stiffness measured with a fix span with the results of the individualised setting we could clearly demonstrate, that not only the absolute values were altered, moreover and more important, the variance within the samples was reduced significantly.

To reach consistent and reliable results, the aspect ratio (span of the lower supports / diameter of the bone at midshaft) of the tested bone should be maximized [30]. By doing so, shear forces are reduced to a minimum and bending as main component of the deflection of bone pronounced. That brings the tested specimen close to fulfilling the ideal beam criteria, which means the least amount of measurement error and the optimal condition for biomechanical characterization of the whole bone. Thus, the span between the lower supports should be increased as much as the bone allows, which is another advantage of an individualised testing protocol and favours results that are more accurate and consistent.

Adapting the biomechanical setup to the bone has been proposed before [9, 31, 32]. By introducing relative sizing (percentage) with respect to the whole bone, another step towards comparability and standardisation was presented in this study. In all specimens the percentage values corresponded well to the natural supporting surfaces of each bone directly supracondylar and directly below the lesser trochanter, which facilitated positioning of the bones on the supporting bars. Precise definition of these sections facilitated radiographic analysis exactly at the given distances and thus favoured correlation with biomechanical data [19].

In general, we reached high and consistent correlations of the BMC assessed by DXA and the pQCT parameters total content and cortical content measured at the shaft with the actual failure load of the bone. In previous studies a strong and positive correlation between biomechanics of bone and microstructural parameters obtained by pQCT and DXA have been reported for femurs of inbred mice [33] and for the human radius [34], both emphasizing the predictive value of the radiologic analysis. The work of Bagi et al. [35] shows similar results to our study. They evaluated cortical microstructural parameters of the unfractured rat femur by μCT, pQCT and DXA and were able to predict cortical bone strength for some of them. Compared to our results, their coefficients of determination (R^2^), regardless of the actual method or parameter, remained relatively low (e.g. correlation between bone strength of the femoral midshaft done by three-point bending and bone mineral density of the whole femur determined by DXA: R^2^ = 0.34; in our study R^2^ = 0.89) [35]). As their biomechanical bending setup implicated a fix span of 15 mm, this might be an argument indicating that better correlations between radiological parameters and biomechanics can be achieved in an individualized setting.

The weight of the testing animal seems to be another factor influencing consistency of results within the trait. In our study, we observed slightly more interindividual differences concerning the failure loads in young and light specimens. Interestingly, intraindividual variance of failure loads (left vs. right) was lowest in this group, whereas mature animals showed only a weak correlation between the left and the right side and relatively large differences in lengths and failure loads between the two bones of one animal. Though not reaching statistical significance in our data, one possible explanation could be that in rats something like handedness evolves during the lifespan, resulting in one favoured side (preferentially used for example as leading leg in climbing) and causing biomechanically relevant differences. In that context, Cunha et al. were recently able to prove paw-preferences and dexterity in behavioural tests of Sprague-Dawley male rats [36]. As previous, biomechanical studies in goats, sheep and dogs did not find relevant right/left differences [37–39], the concept of handedness in animals seems to be underestimated in the past. This might be an issue to investigate or rethink in the future. Concerning the stiffness, we observed the same behaviour but less clearly pronounced. With respect to our data, small rats should potentially be more suitable for normalized examinations (failure load of the experimental side as ratio to healthy side).

The fixation method we used potentially limits transferability of the data [40, 41]. Although Linde and Sorensen did not find relevant changes in the biomechanical behaviour of human trabecular bone specimens stored in 70% methanol for 100 days [42] and Mick et al. stated no differences for ultimate bending of human cortical specimens after rehydration due to formalin or ethanol-fixation (96%) compared to fresh frozen bones [43], we do not know the relevance of possible modifications caused by our treatment protocol. Anyway, as the comparison between the groups is more significant than the absolute values and all bones were treated equally, this systematic failure should not affect the main deductions drawn by our study.

## Conclusions

Knowledge on natural fluctuation within a test population influences the sample size and limits the verifiability of any experimental result [4]. The given study focuses a systematic assessment of the biomechanical and morphological characteristics of femurs of the male Wistar-rat to improve accurate and unbiased sample size calculations in future experiments. Ruling out that functional relevance of morphological variations causes a methodological gap to obtain a level of significance, we furthermore aimed to close this gap with a methodological variation of biomechanical testing by respecting the individual length of every specimen. Furthermore, our results show that relevant, intraindividual side-differences potentially relate to dexterity in rat femurs of heavy (old) animals. Nevertheless, using paired specimens seems applicable, especially in small animals. By adapting the setting of the biomechanical testing to the individual length of the bone, interindividual variation could be reduced significantly. To achieve consistent results and to compare different studies, an individualized setting of biomechanical testing would be beneficial.

## Additional file

Additional file 1: Supplemental data sheets. (PPTX 35 kb)

## Abbreviations

AP: Anterioposterior
BMC: Bone mineral content
BMD: Bone mineral density
DXA: Dual energy X-ray absorptiometry
pQCT: Peripheral quantitative computed tomography
