# Supplementary material for: Improving results in rat fracture models: enhancing the efficacy of biomechanical testing by a modification of the experimental setup
Source: BMC Musculoskelet Disord. 2018 Jul 19;19:243. doi: 10.1186/s12891-018-2155-y (PMC6053723; doi:10.1186/s12891-018-2155-y)
Supplement: Supplementary file 1 — Supplemental data sheets. (PPTX 35 kb) [file 12891_2018_2155_MOESM1_ESM.pptx]

## Slide 1
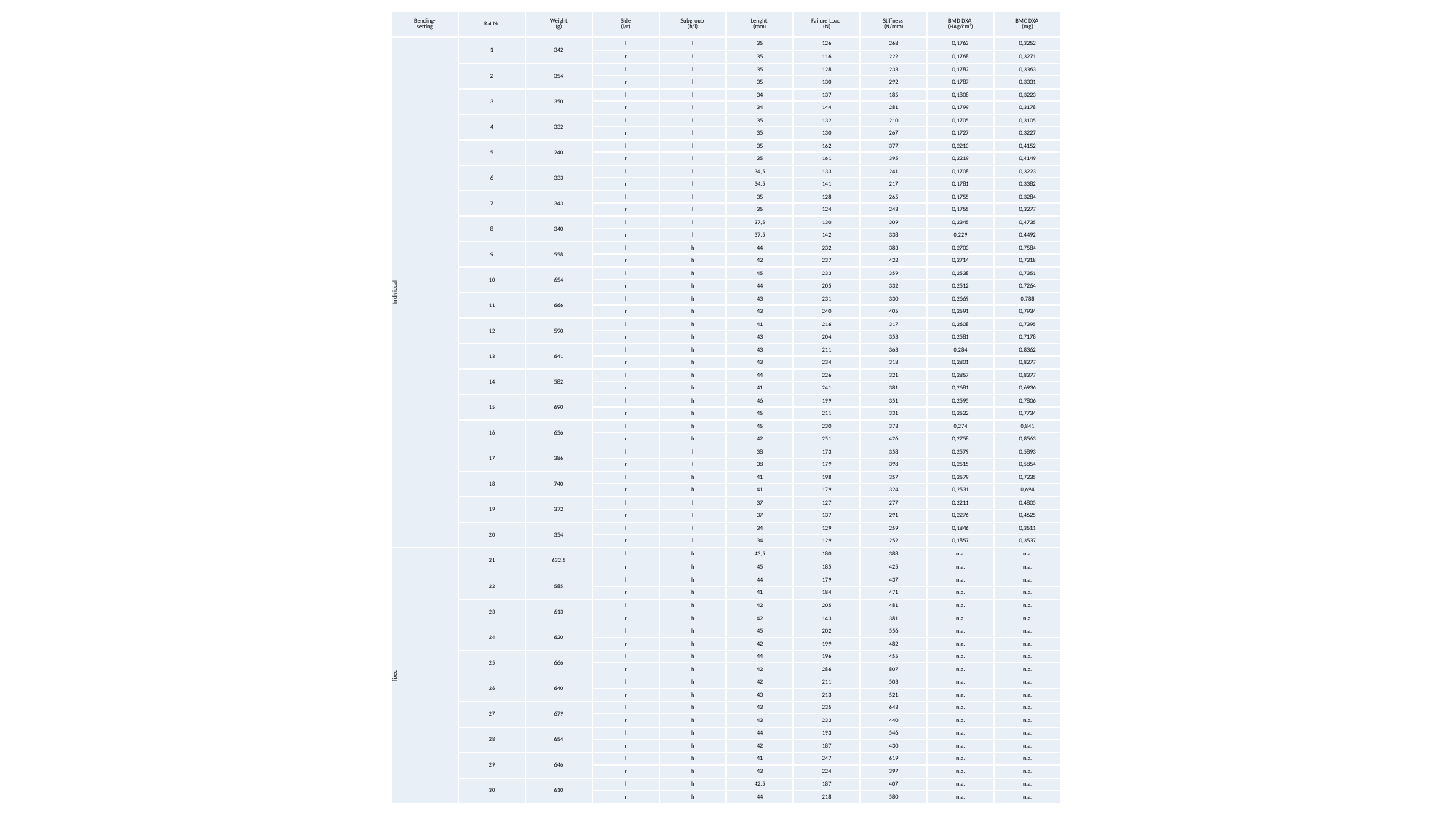

| Bending- setting | Rat Nr. | Weight (g) | Side (l/r) | Subgroub (h/l) | Lenght (mm) | Failure Load (N) | Stiffness (N/mm) | BMD DXA (HAg/cm³) | BMC DXA (mg) |
| --- | --- | --- | --- | --- | --- | --- | --- | --- | --- |
| Individual | 1 | 342 | l | l | 35 | 126 | 268 | 0,1763 | 0,3252 |
| | | | r | l | 35 | 116 | 222 | 0,1768 | 0,3271 |
| | 2 | 354 | l | l | 35 | 128 | 233 | 0,1782 | 0,3363 |
| | | | r | l | 35 | 130 | 292 | 0,1787 | 0,3331 |
| | 3 | 350 | l | l | 34 | 137 | 185 | 0,1808 | 0,3223 |
| | | | r | l | 34 | 144 | 281 | 0,1799 | 0,3178 |
| | 4 | 332 | l | l | 35 | 132 | 210 | 0,1705 | 0,3105 |
| | | | r | l | 35 | 130 | 267 | 0,1727 | 0,3227 |
| | 5 | 240 | l | l | 35 | 162 | 377 | 0,2213 | 0,4152 |
| | | | r | l | 35 | 161 | 395 | 0,2219 | 0,4149 |
| | 6 | 333 | l | l | 34,5 | 133 | 241 | 0,1708 | 0,3223 |
| | | | r | l | 34,5 | 141 | 217 | 0,1781 | 0,3382 |
| | 7 | 343 | l | l | 35 | 128 | 265 | 0,1755 | 0,3284 |
| | | | r | l | 35 | 124 | 243 | 0,1755 | 0,3277 |
| | 8 | 340 | l | l | 37,5 | 130 | 309 | 0,2345 | 0,4735 |
| | | | r | l | 37,5 | 142 | 338 | 0,229 | 0,4492 |
| | 9 | 558 | l | h | 44 | 232 | 383 | 0,2703 | 0,7584 |
| | | | r | h | 42 | 237 | 422 | 0,2714 | 0,7318 |
| | 10 | 654 | l | h | 45 | 233 | 359 | 0,2538 | 0,7351 |
| | | | r | h | 44 | 205 | 332 | 0,2512 | 0,7264 |
| | 11 | 666 | l | h | 43 | 231 | 330 | 0,2669 | 0,788 |
| | | | r | h | 43 | 240 | 405 | 0,2591 | 0,7934 |
| | 12 | 590 | l | h | 41 | 216 | 317 | 0,2608 | 0,7395 |
| | | | r | h | 43 | 204 | 353 | 0,2581 | 0,7178 |
| | 13 | 641 | l | h | 43 | 211 | 363 | 0,284 | 0,8362 |
| | | | r | h | 43 | 234 | 318 | 0,2801 | 0,8277 |
| | 14 | 582 | l | h | 44 | 226 | 321 | 0,2857 | 0,8377 |
| | | | r | h | 41 | 241 | 381 | 0,2681 | 0,6936 |
| | 15 | 690 | l | h | 46 | 199 | 351 | 0,2595 | 0,7806 |
| | | | r | h | 45 | 211 | 331 | 0,2522 | 0,7734 |
| | 16 | 656 | l | h | 45 | 230 | 373 | 0,274 | 0,841 |
| | | | r | h | 42 | 251 | 426 | 0,2758 | 0,8563 |
| | 17 | 386 | l | l | 38 | 173 | 358 | 0,2579 | 0,5893 |
| | | | r | l | 38 | 179 | 398 | 0,2515 | 0,5854 |
| | 18 | 740 | l | h | 41 | 198 | 357 | 0,2579 | 0,7235 |
| | | | r | h | 41 | 179 | 324 | 0,2531 | 0,694 |
| | 19 | 372 | l | l | 37 | 127 | 277 | 0,2211 | 0,4805 |
| | | | r | l | 37 | 137 | 291 | 0,2276 | 0,4625 |
| | 20 | 354 | l | l | 34 | 129 | 259 | 0,1846 | 0,3511 |
| | | | r | l | 34 | 129 | 252 | 0,1857 | 0,3537 |
| fixed | 21 | 632,5 | l | h | 43,5 | 180 | 388 | n.a. | n.a. |
| | | | r | h | 45 | 185 | 425 | n.a. | n.a. |
| | 22 | 585 | l | h | 44 | 179 | 437 | n.a. | n.a. |
| | | | r | h | 41 | 184 | 471 | n.a. | n.a. |
| | 23 | 613 | l | h | 42 | 205 | 481 | n.a. | n.a. |
| | | | r | h | 42 | 143 | 381 | n.a. | n.a. |
| | 24 | 620 | l | h | 45 | 202 | 556 | n.a. | n.a. |
| | | | r | h | 42 | 199 | 482 | n.a. | n.a. |
| | 25 | 666 | l | h | 44 | 196 | 455 | n.a. | n.a. |
| | | | r | h | 42 | 286 | 807 | n.a. | n.a. |
| | 26 | 640 | l | h | 42 | 211 | 503 | n.a. | n.a. |
| | | | r | h | 43 | 213 | 521 | n.a. | n.a. |
| | 27 | 679 | l | h | 43 | 235 | 643 | n.a. | n.a. |
| | | | r | h | 43 | 233 | 440 | n.a. | n.a. |
| | 28 | 654 | l | h | 44 | 193 | 546 | n.a. | n.a. |
| | | | r | h | 42 | 187 | 430 | n.a. | n.a. |
| | 29 | 646 | l | h | 41 | 247 | 619 | n.a. | n.a. |
| | | | r | h | 43 | 224 | 397 | n.a. | n.a. |
| | 30 | 610 | l | h | 42,5 | 187 | 407 | n.a. | n.a. |
| | | | r | h | 44 | 218 | 580 | n.a. | n.a. |
